# Injectable mechanical pillows for attenuation of load-induced post-traumatic osteoarthritis

**DOI:** 10.1093/rb/rbz013

**Published:** 2019-04-22

**Authors:** Derek T Holyoak, Tibra A Wheeler, Marjolein C H van der Meulen, Ankur Singh

**Affiliations:** 1 Meinig School of Biomedical Engineering, Cornell University, Ithaca, NY, USA; 2 Sibley School of Mechanical and Aerospace Engineering, Cornell University, Ithaca, NY, USA; 3 Research Division, Hospital for Special Surgery, New York, NY, USA; 4 Englander Institute for Precision Medicine, Weill Cornell Medical College, Cornell University, New York, NY, USA

**Keywords:** hydrogel, osteophyte, biomaterials, drug delivery, cartilage, inflammation

## Abstract

Osteoarthritis (OA) of the knee joint is a degenerative disease initiated by mechanical stress that affects millions of individuals. The disease manifests as joint damage and synovial inflammation. Post-traumatic osteoarthritis (PTOA) is a specific form of OA caused by mechanical trauma to the joint. The progression of PTOA is prevented by immediate post-injury therapeutic intervention. Intra-articular injection of anti-inflammatory therapeutics (e.g. corticosteroids) is a common treatment option for OA before end-stage surgical intervention. However, the efficacy of intra-articular injection is limited due to poor drug retention time in the joint space and the variable efficacy of corticosteroids. Here, we endeavored to characterize a four-arm maleimide-functionalized polyethylene glycol (PEG-4MAL) hydrogel system as a ‘mechanical pillow’ to cushion the load-bearing joint, withstand repetitive loading and improve the efficacy of intra-articular injections of nanoparticles containing dexamethasone, an anti-inflammatory agent. PEG-4MAL hydrogels maintained their mechanical properties after physiologically relevant cyclic compression and released therapeutic payload in an on-demand manner under *in vitro* inflammatory conditions. Importantly, the on-demand hydrogels did not release nanoparticles under repetitive mechanical loading as experienced by daily walking. Although dexamethasone had minimal protective effects on OA-like pathology in our studies, the PEG-4MAL hydrogel functioned as a mechanical pillow to protect the knee joint from cartilage degradation and inhibit osteophyte formation in an *in vivo* load-induced OA mouse model.

## Introduction

Osteoarthritis (OA) of the knee joint is a degenerative biomechanical disease that affects ∼27 million individuals in the USA and is the leading cause of disability in the elderly population [1–3]. Three primary characteristics of OA are cartilage degradation, osteophyte formation and synovial inflammation [4]. During the development of OA, enzymes such as aggrecanases and matrix metalloproteinase (MMP)-3 and MMP-13, degrade the cartilage matrix and are elevated in the joint space [5–9]. Post-traumatic osteoarthritis (PTOA) is a specific form of OA associated with joint damage that develops after mechanical trauma to the joint [10]. For example, 14 years after an anterior cruciate ligament (ACL) tear, 78% of young adults develop radiographic changes and 40% have radiographic OA in the injured knee [11]. This degeneration of the joint is rapid and irreversible. Once damage is evident radiographically, the progression cannot be reversed. Therefore, therapeutic interventions immediately post-injury are needed to prevent the development of load-induced OA.

Controlled mechanical loading models used to study bone adaptation and signaling [12–14] provide an opportunity to define the role of mechanical loading on cartilage and whole joint pathology [15]. We have developed a non-invasive, load-induced murine model of OA using controlled cyclic tibial compression [16]. This model provides controlled, consistent kinematics in the knee joint without ligament injury [17] and recapitulates OA-like pathology after 1, 2 and 6 weeks of daily loading at a peak load of 9N in mice [16]. We have previously reported load-level-dependent intrinsic alterations in bone and articular cartilage composition. In addition, the bone and cartilage changes present after a single bout of loading in this tibial loading model mimicked PTOA [18] and is a focus of the current study. A single 5-min dose of 9N-peak cyclic loading did not induce macroscopic cartilage damage immediately after loading (0 week), but rather initiated a cellular response that degrades the cartilage and alters bone morphology by 2 weeks post-loading. This load-induced model of OA allows us to explore disease mechanisms and treatment options targeted at specific tissues in the joint, without the confounding effects of invasive procedures.

We hypothesized that intra-articular injection of a mechanical pillow that can withstand routine forces in weight-bearing joints of mouse and release therapeutics in an on-demand fashion will provide a cushioning effect and attenuate the development of load-induced OA. Both cushioning and delivery of anti-inflammatory therapeutics are expected to alleviate pain and slow the progression of damage in the joint to prevent PTOA. Ultimately, we anticipate that the reduced joint pathology will attenuate load-induced OA pathology and potentially permit regeneration of the cartilage.

In comparison to other treatments, such as oral drugs or local anesthetics, intra-articular (IA) injections of anti-inflammatory drugs are advantageous for OA treatment because of the localized drug delivery into the diseased joint and reduced risk of side effects. However, drugs are rapidly cleared from the joint space into the lymphatics at ∼0.04 ml/min corresponding to a turnover of as short as ∼1 h [19, 20]. The rapid clearance reduces the availability of drugs over time, requiring their frequent administration (e.g. weekly for up to 6 weeks) [21, 22]. Repeated injections are an obvious disadvantage as they cause discomfort and pain and can also lead to infections. To overcome the limitation of poor drug retention in knee joints, recent focus has shifted towards using emulsion or biomaterials-based microparticles, nanoparticles and hydrogels that remain in the joint longer and release drug over time [22–27]. Among these bioengineered approaches, injectable hydrogels provide a unique multi-modal drug delivery platform that can encapsulate nanoparticles within themselves [28–30]. However, developing a hydrogel platform that can withstand PTOA pathology requires specific design considerations. Ideally, an IA hydrogel delivery system should withstand routine forces in weight-bearing joints (up to 40% strain in cartilage) [31], maintain constant volume for long durations under hydrolytic conditions, and release therapeutics in the presence of degradative proteases, such as aggrecanases and collagenases (e.g. MMP-3 and MMP-13).

Here, we developed and characterized a ‘mechanical pillow’ using a four-arm maleimide-functionalized polyethylene glycol (PEG-4MAL) hydrogel with encapsulated polymeric nanoparticles. We and others have previously reported PEG-4MAL hydrogels crosslinked to protease-degradable crosslinkers [32–35]. We establish the mechanical stability of hydrogels under daily cyclic compression conditions and hydrolytic conditions and controlled proteolytic degradation under elevated levels of MMPs. We demonstrate that the hydrogels retained swelling over a prolonged period *in vitro*. Finally, *in vivo* data prove that the gels protected the joint from cartilage degradation and inhibited osteophyte formation in a mouse model of load-induced PTOA.

## Materials and methods

### Synthesis of PEG-4MAL-based mechanical pillows

PEG-4MAL hydrogels were synthesized, as reported by others and us [32–35]. PEG-4MAL was obtained with >90% purity (Laysan Bio, Inc., Arab, AL, USA). Briefly, for the *in vitro* dynamic cyclic compression and swelling experiments, 50 μl droplets of synthetic hydrogels were fabricated. The hydrogels were composed of 2.5, 5, 10 or 20% (w/v) PEG-4MAL macromers reconstituted in 1% HEPES buffer with a pH of 7.4. PEG-4MAL was crosslinked with non-degradable dithiothreitol (DTT) crosslinker at a 1:1.5 PEG-4MAL:crosslinker molar ratio. Each hydrogel contained fluorescent fluorescein isothiocyanate (FITC) polystyrene particles (5 μm diameter, Duke Scientific Corporation) suspended in crosslinker solution at a concentration of 2000 particles/μl to mimic therapeutic particles, resulting in ∼100 000 particles per 50 µl hydrogel. To prepare hydrogel droplets, 25 μl of macromer solution was placed in the middle of a nontreated 24-well plate, 25 μl of particle-containing cross-linker solution was injected into the initial droplet, mixed by pipetting up and down and cured at 37°C for 15–20 min inside a cell culture incubator for complete crosslinking. Then, phosphate-buffered saline (PBS) was added to each well to cover and hydrate the hydrogels (*n* = 6/group).

For the *in vitro* collagenase experiments, 10 μl hydrogels with 10% (w/v) PEG-4MAL macromer in HEPES buffer were fabricated. PEG-4MAL was combined with non-DTT crosslinker and/or MMP-degradable GCRDVPMSMRGGDRCG peptides (VPM) (AAPPTec, LLC, >90% purity). Three crosslinker combinations were evaluated to control the degradability of the hydrogel: 100% DTT and 0% VPM, 50% DTT and 50% VPM (1:1 molar ratio) or 0% DTT and 100% VPM. The PEG-4MAL: crosslinker ratio was maintained at 1:1.5 molar ratio in all three solutions. In these studies, each hydrogel contained fluorescent FITC polystyrene particles (200 nm, Thermo Fisher Scientific) suspended in the crosslinker solution at ∼14 000 particles/μl concentration, resulting in ∼140 000 particles per 10 µl hydrogel. Hydrogel droplets were prepared and cured at 37°C for 15–20 min inside a cell culture incubator for complete crosslinking (*n* = 4/group). The particle size in these collagenase studies was aligned with our *in vivo* studies that used ∼200 nm particles.

For the *in vivo* experiments, due to limited space in the mouse joint [36], 2 μl hydrogels with 10% (w/v) PEG-4MAL concentration in HEPES buffer were used. Based on the *in vitro* results, the crosslinker solution was 50% DTT and 50% VPM (*n* = 5/group).

### Particle release studies

#### Dynamic cyclic mechanical compression of hydrogels

**Figure 1:**
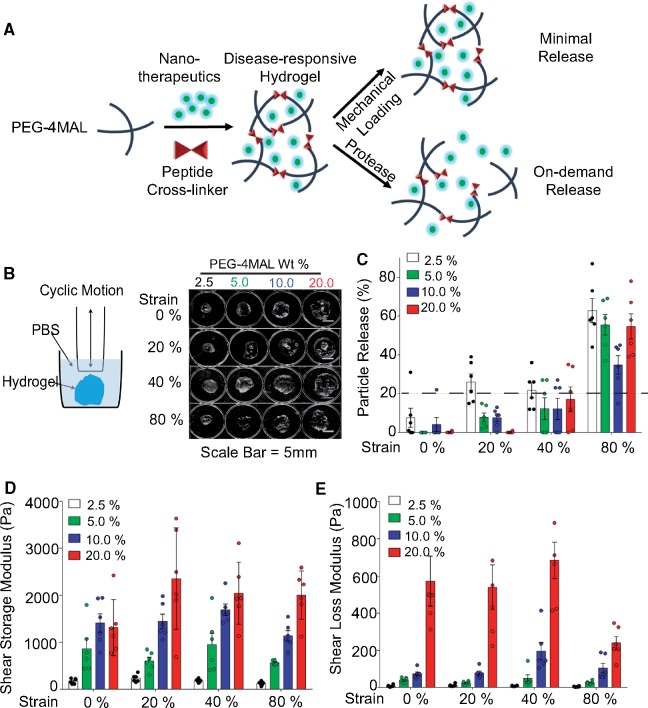
PEG-4MAL hydrogels with nanoparticles form mechanical pillows that retained therapeutics and maintained viscoelastic properties after *in vitro* dynamic cyclic compression. (**A**) Schematic representing the overall ‘mechanical pillow’ concept of maintaining the integrity and retaining therapeutic cargo under daily mechanical loading and releasing drug under protease-rich conditions. (**B**) Schematic of a custom-made bioreactor (left) and images of the overall structure hydrogels after cyclic compression at 80% strain levels (right). (**C**) Particle release in all hydrogel groups across 0–80% strain. The dotted line represents <20% release. Up to 40% strain represents physiological daily repetitive loading in a human and 80% strain represents injurious mechanical loading. (**D, E**) Scatter plots showing maintenance of viscoelastic properties of the mechanical pillows with mechanical loading. Shear storage (**D**) and loss moduli (**E**) increased with increased PEG-4MAL weight percentages. *n* = 6/group.

Immediately after fabrication, hydrogels were placed in PBS in a 24-well plate. Hydrogels underwent unconfined cyclic compression at 37°C with a custom-built, displacement-controlled bioreactor, as described previously [37] (Fig. 1B). Cyclic compression was applied at strain levels of 0, 20, 40 and 80% for 10 000 cycles at 1 Hz. Particle release was measured in the supernatant from each well following loading of the hydrogel. The supernatant was analysed for particles by flow cytometry (BD Accuri C6 flow cytometer, BD Biosciences). Then the resulting particle counts were gated to output the number of particles in the FITC fluorescent range (Accuri software).

#### Protease-mediated degradation of hydrogels

Hydrogels were fabricated in a 96-well plate and subjected to 200 μl collagenase (Type 1, Worthington) at 0 U/ml, 1 U/ml or 10 U/ml immediately after fabrication. After 3 h of collagenase exposure, particle release was measured in the supernatant from each well by flow cytometry. Then resulting particle counts were gated to output the number of particles in the FITC fluorescent range (Accuri software). The residual hydrogels also were imaged using fluorescent imaging (IVIS-200, Xenogen). Next, hydrogels were resuspended in PBS for 21 h. Then, hydrogels were reintroduced to collagenase for another 3 h, and the supernatant was collected and analysed again. This process was repeated daily for 4 days.

### Rheological properties of hydrogels-nanoparticle combination

The storage and loss moduli of each hydrogel were measured using a Discovery Hybrid Rheometer (TA Instruments, New Castle, DE, USA). A parallel plate geometry of 8 mm diameter was used. The oscillatory degree of rotation (5°), frequency (0.1 Hz) and temperature (37°C) were constant throughout all tests. All samples underwent one cycle of preconditioning. We recorded shear storage (G') and loss (G'') moduli as a function of time [38, 39] and calculated average moduli over four cycles for each hydrogel.

### Swelling ratio

Immediately after fabrication, the initial hydrogel weight was measured. Then, the hydrogels were incubated in 1 ml PBS in a 24-well plate at 37°C (*n* = 6/group). For the next 15 days, the PBS was removed, the weights of the swollen hydrogels were measured, and the wells with hydrogels were replenished with 1 ml PBS. The swelling ratio was determined according to the following equation:

$$\text{Swelling}\;\text{ratio}=\frac{\text{Swollen}\;\text{weight}}{\text{Initial}\;\text{weight}}\times 100$$

### DEX nanoparticle fabrication

Poly(lactic-co-glycolic acid) (PLGA) nanoparticles (NPs) were synthesized based on established protocols with modifications [40]. PLGA (30 mg) and dexamethasone (DEX) (10 mg) were simultaneously dissolved in 1 ml acetone. The solution was vortexed and sonicated for 7 min to create a water-in-oil emulsion. This emulsion was added to a 6 ml solution of 2% pol(vinyl alcohol) (PVA) in PBS and sonicated for 7 min. The resulting solution was stirred at 900 rpm for 4 h, followed by stirring at 900 rpm for 1 h in a vacuum desiccator. The nanoparticles were centrifuged at 20 000 *g*, resuspended in PBS and sonicated for 30 s. The wash step was repeated three times. The nanoparticle size was 203 ± 7 nm, as quantified by dynamic light scattering (Zetasizer, Brookhaven).

### Injection of formulations in the knees of an in vivo mouse model of PTOA

#### Treatment groups

A 26-week-old C57Bl/6 male mice (*n* = 5/group; Jackson Laboratory) were purchased and acclimated. PTOA was induced in mouse knees using cyclic tibial compression, as we reported earlier [12, 18] and adopted by others [41]. To determine the therapeutic efficacy of the hydrogel system, mice were divided into five treatment groups: (i) saline; (ii) bolus DEX; (iii) DEX-loaded PLGA nanoparticles; (iv) hydrogel with DEX-loaded PLGA nanoparticles; and (v) hydrogel alone. Two weeks after treatment, mice were euthanized, and knee joints were harvested and fixed in 4% paraformaldehyde overnight at 4°C. All studies were performed in compliance with the Institutional Animal Care and Use Committee (IACUC) at Cornell University.

#### In vivo mechanical loading

A single session of cyclic compression was applied to the tibia to mimic PTOA. With the mice under general anaesthesia (2% isoflurane, 1.0 l/min, Webster), loading was applied to the left or right tibia at a 9.0N-peak load magnitude for 1200 cycles at 4 Hz. Contralateral limbs served as controls.

#### Intra-articular injections

Approximately 48 h after the single loading session, both mouse knee joints received 2 μl intra-articular injections of hydrogels with 10% (w/v) PEG-4MAL in HEPES buffer. The crosslinker solution was composed of 50% DTT and 50% VPM (*n* = 5/group). The PEG-4MAL: crosslinker molar ratio was 1:1.5. With mice under general anaesthesia (2% isoflurane, 1.0 l/min), a 2 mm skin incision was made with a Number 15 blade to expose the patellar tendon. Thereafter, 2 µl of formulation was injected into the joint space using a Hamilton syringe. The injection location was immediately medial to the patellar tendon. Skin incisions were closed with 6-0 prolene suture (8706H, Ethicon). Buprenorphine (0.1 mg/kg, Reckitt) was administered for two days post-operatively. For bolus injections, DEX (5 mg/ml, Sigma D1881) was dissolved in PBS [42, 43]. The final concentration of DEX in the nanoparticles was approximated to be 0.3 mg DEX per mg PLGA, based on prior studies on dexamethasone in 200 nm PLGA particles [44] and other PLGA formulations by us [29, 30, 45]. The hydrogel group with DEX-loaded nanoparticles contained ∼10% nanoparticles, and therefore, the final concentration of DEX in pillows was ∼1.8 µg per 2 µl gel injected into the joint. To match this dose, the nanoparticle only group was diluted in PBS for a final concentration of ∼1.8 µg DEX per 2 µl nanoparticles injected into the joint. In contrast, the bolus dose was 10 μg DEX per knee.

### Cartilage degradation

Mechanically loaded and non-loaded control mouse knee joints were harvested and decalcified in 10% ethylenediaminetetraacetic acid (EDTA) for 2 weeks and embedded in paraffin. Paraffin blocks were sectioned at a thickness of 6 µm from posterior to anterior using a rotary microtome (Leica RM2255, Wetzlar, Germany). Cartilage morphology in the tibial plateau was assessed using Safranin O/Fast Green-stained sections at 90 μm intervals throughout the joint. The OARSI scoring system was used to assess structural cartilage damage [46]. Scores were measured in the medial tibial plateau and averaged across all sections of each limb.

### Osteophyte formation

Safranin-O/fast green-stained sections were examined for osteophyte formation on the medial aspect of the tibia. Medial osteophytes from three representative sections in the joint (posterior, middle and anterior) were analysed. The medial-lateral width of the osteophyte was measured, defined as the distance between the medial end of the epiphysis and the end of the osteophyte [47]. Overall osteophyte size is reported as the mean width of the three sections.

### Statistical analyses

To analyse the effects of cyclic compression on cartilage mechanical properties and particle release, a two-way analysis of variance (ANOVA) was used, with PEG-4MAL weight percentage and loading condition as variables. To analyse the effects of collagenase exposure on the hydrogels, a three-way ANOVA was used with the hydrogel crosslinker, collagenase concentration, and duration as variables. For *in vivo* joint morphology, a linear mixed-effects model was used to assess the effects of loading, the effects of hydrogel vs. no hydrogel nested by loading, and the effects of DEX vs. no DEX nested by loading and the presence of hydrogel. Individual data are presented as mean ± standard deviation. Alpha levels for statistical significance were set to *P* = 0.05.

## Results and discussion

### Hydrogels maintained mechanical properties after dynamic cyclic compression

We hypothesized that PEG-4MAL weight percentage in an engineered hydrogel system will regulate whether the injectable drug delivery system can withstand routine forces in weight-bearing joints and function as a mechanical pillow. We applied cyclic compression on freshly formed hydrogels at strain levels of 0, 20, 40 and 80% for 10 000 cycles at 1 Hz. Joint loading can result in cartilage strains up to 40% during normal activity and 80% during injurious activity, such as a traumatic joint injury [31]. All hydrogel groups maintained their viscoelastic properties after dynamic cyclic compression. Hydrogels maintained overall structure even after cyclic compression at 80% strain levels (Fig. 1B). All hydrogel groups released minimal therapeutics (≤20%) during typical daily activities, modelled as cyclic loads of 20% or 40% strain (Fig. 1C). However, with 80% strain, approximately half of the encapsulated particles were released after cyclic compression. Depending on the location of the hydrogel within the joint and the degree of physical activity, physiological strains in cartilage could result in 80% strain levels and cause particle release. About 10% PEG-4MAL hydrogels released ∼20% fewer particles compared with other groups after cyclic compression at 80% strain. Hydrogels with higher PEG-4MAL weight percentage had greater shear storage and loss moduli, which can be attributed to crosslinking density of the hydrogels [48, 49]. At all PEG-4MAL weight percentages, hydrogels withstood mechanical loading without changes in shear storage and loss moduli after cyclic compression (*P* = 0.996) (Fig. 1D and E).

The viscoelastic properties of these hydrogels result from covalently crosslinked polymer chains that are also physically entangled with each other. At lower strains, the elastic networks dominate, whereas at higher strains significant plastic deformation occurs, as shown by the data. The elastic and plastic properties change with polymer concentration, leading to structural inhomogeneity. *In situ* forming, fast reaction Michael type addition hydrogels can exhibit heterogeneous structure [50]. In this chemical reaction, the macromer PEG-4MAL reacts with thiols. Because the nanoparticles lack a suitable chemical group to bind to the electron-deficient ends in the maleimide, we do not expect covalent or chemical interactions. However, we anticipate physical interactions, such as entanglements [30]. Based on the elastic and plastic contribution of the network along with structural inhomogeneity, the 10% PEG-4MAL hydrogel can lead to lower particle release compared to other formulations. In addition, the dynamic mechanical loading can influence the pore size of the hydrogel network, which also governs nanoparticle release from the network.

### Hydrogel swelling ratio increased with PEG-4MAL weight percentage

**Figure 2:**
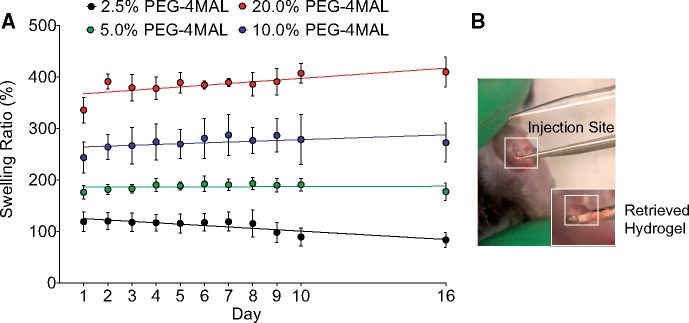
PEG-4MAL hydrogels with nanoparticles maintained swelling *in vitro* and retained integrity *in vivo*. (**A**) Swelling ratio of PEG-4MAL hydrogels with varying weight percentages over time up to 2 weeks (*n* = 6/group). (**B**) Image of hydrogel retrieved from the intra-articular injection site after 3 weeks post-injection and being subjected to daily activities of the mouse.

We next hypothesized that PEG-4MAL weight percentage will regulate the swelling and size of hydrogels. We sought to balance hydrogel swelling and mechanical properties to ensure minimal swelling in the limited space of the joint cavity. All gels had similar weights at the initial time point, but after 16 days, higher PEG-4MAL weight percentages resulted in larger swelling ratios (Fig. 2A). The majority of swelling occurred within the first 24 h after crosslinking before reaching equilibrium, consistent with previous reports [48].

Although a primary goal was to minimize hydrogel swelling, the balance between mechanical integrity and swelling led to the decision to use 10% (w/v) PEG-4MAL hydrogel for subsequent studies. The 2.5% PEG-4MAL hydrogels demonstrated the least swelling compared to any other hydrogel formulation. However, the 2.5% PEG-4MAL hydrogels had the weakest mechanical properties (Fig. 1D and E). Ultimately, the 10% PEG-4MAL hydrogels withstood cyclic compression at 80% strain most optimally (i.e. released fewest particles) compared to other formulations. In addition, the 10% PEG-4MAL hydrogels had lower swelling ratios than 20% PEG-4MAL hydrogels. Swelling is not expected to be as large of an issue for *in vivo* mouse studies because the joint does not contain excessive fluid. Therefore, the 10% PEG-4MAL hydrogel was used in the remainder of this study. We confirmed the *in vivo* integrity of the 10% PEG-4MAL hydrogel by injecting into the knee joint and retrieving the hydrogel after 3 weeks (Fig. 2B). Retrieval of the hydrogel demonstrated that the hydrogel resided near the fat pad in the mouse knee joint. Furthermore, the harvested hydrogels were not degraded or markedly swollen, demonstrating the stability of these mechanical pillows under normal loading conditions.

### Hydrogels released encapsulated nanoparticles in an on-demand manner

**Figure 3:**
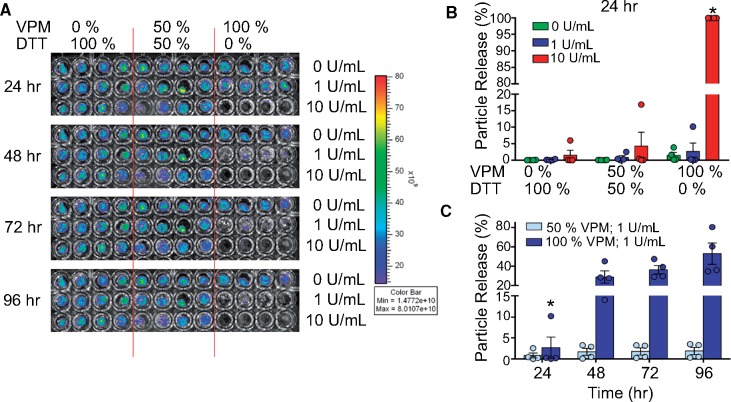
PEG-4MAL Hydrogels release therapeutics in a formulation and protease-dependent manner. (**A**) Fluorescent particle release over time imaged by IVIS. (**B**) Flow cytometry analysis of nanoparticle release from PEG-4MAL hydrogels when exposed to collagenase as a function of protease concentration and crosslinker composition (percentage of DTT vs. VPM). (**C**) Scatter plot comparing particle release over time of hydrogels fabricated with 50% VPM vs. 100% VPM hydrogels. All groups used *n* = 4 hydrogels; *P < 0.05 compared to all other groups.

OA pathology manifests increased levels of MMP-3 and MMP-13 in the joint space during early and late stages of OA [51]. The suppression of inflammation should reduce MMP levels; however, with time OA flares will again increase the MMP levels. A strategic solution to this limitation would be the development of MMP-responsive hydrogels that can release therapeutics when spiked with proteases. We used previously established MMP-degradable PEG-4MAL hydrogels to release drug or nanoparticles in an on-demand manner [32–34]. Particle release from the hydrogel was dependent on the proportion of MMP-degradable crosslinker, the concentration of collagenase and the number of days hydrogels were exposed to proteases (Fig. 3). Within the first 24 h, non-degradable hydrogels with 0% VPM peptide and 100% DTT crosslinker demonstrated minimal or no particle release upon *in vitro* collagenase exposure at 1 or 10 U/ml concentrations. In contrast, ∼5% of particles were released from hydrogels formulated with 50% VPM peptide and 50% DTT crosslinker, when exposed to 10 U/ml collagenase. Hydrogels fabricated with 100% VPM demonstrated ∼100% particle release with 10 U/ml collagenase (*P* < 0.0001; Fig. 3B), and no significant differences were seen between groups with various crosslinker ratios at 0 or 1 U/ml collagenase exposure. Maximum particle release occurred after the first collagenase exposure and then remained relatively constant after repeated further exposure (*P* < 0.0001). At 1 U/ml of collagenase, the 50% VPM hydrogels released significantly fewer particles compared to the 100% VPM hydrogels over multiple collagenase exposures (48, 72 and 96 h, *P* = 0.0023, *P* = 0.0001, *P* < 0.0001, respectively; Fig. 3C). Collectively, our results indicate that the PEG-4MAL hydrogels with nanoparticles did not release payload under normal levels of daily mechanical loading but instead under the effect of proteases, and as a function of protease concentration, mimicking an on-demand drug release system.

### Mechanical pillows attenuated in vivo load-induced cartilage degradation and osteophyte formation

We hypothesized that load-induced OA will be attenuated *in vivo* by an engineered hydrogel system that can withstand routine forces in weight-bearing joints, provide a cushioning effect and release therapeutics in an on-demand fashion. To prove our hypothesis, we used our established *in vivo* mouse model of load-induced OA.

**Figure 4:**
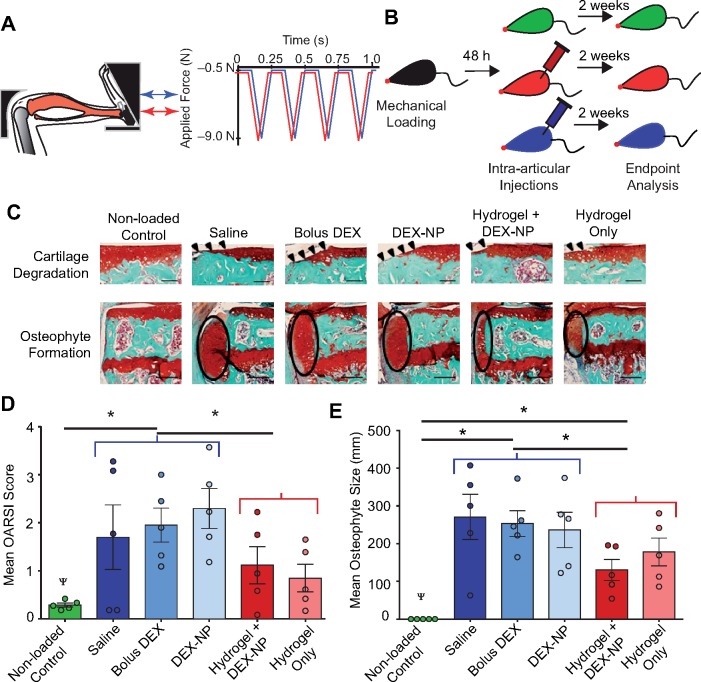
Injectable mechanical pillows attenuated *in vivo* cartilage damage and osteophyte formation following the development of load-induced osteoarthritis. (**A**) Mouse tibial cyclic compression model. Schematic of tibia positioned in loading device, ready for *in vivo* axial loading to be applied. (**B**) Schematic of the duration of loading, intra-articular injections, and end-point analysis. The five injection formulations were saline, bolus DEX (5 mg/ml in PBS), DEX-loaded PLGA nanoparticles (8 mg/mL), hydrogel with DEX-loaded PLGA nanoparticles and hydrogel alone. Hydrogel groups were 10% w/v PEG-4MAL with 1:1 DTT:VPM ratio. The hydrogel group with DEX-nanoparticles was ∼10% nanoparticles. (**C**) Safranin O—fast green-stained images of the non-loaded vs. loaded limbs (peak load 9.0N), indicating cartilage erosion (arrowheads) and osteophyte (ellipses) after 2 weeks of loading in the posterior aspect of the medial tibial Plateau. (**D**) The mean OARSI scores of cartilage in the medial tibial Plateau and (**E**) mean medial-lateral width of the osteophyte from three representative sections in the joint (posterior, middle and anterior). *n* = 5 mice/group. Red groups = non-hydrogel injections; blue groups = hydrogel-containing injections. ^Ψ^*P* < 0.05 for loading; and *P < 0.05 for hydrogel vs. no hydrogel nested by loading. Cartilage scale bars = 100 μm. Osteophyte scale bars = 200 μm.

Approximately 48 h after the single loading session (Fig. 4A), both mouse knee joints received intra-articular injections of 2 μl hydrogels with 10% PEG-4MAL crosslinked with 50% VPM and 50% DTT solution (*n* = 5/group; Fig. 4B). Two weeks after the single bout of cyclic compression and the hydrogel injection, loaded joints in all groups had cartilage degradation and osteophyte formation on the medial tibial plateau (Fig. 4C). In general, loading induced cartilage erosion extending to the tidemark in the posteromedial aspect of the tibial plateau. When we compared the therapeutic efficacy of hydrogel-containing vs. non-hydrogel-containing formulations in the joint environment with load-induced OA, the non-hydrogel formulations had increased OARSI scores compared to control limbs (*P* = 0.0037; Fig. 4C and D). Injections of bolus DEX or DEX-loaded nanoparticles did not have beneficial effects on any load-induced tissue changes. In contrast, cartilage damage was lower in loaded limbs that received injectable hydrogel alone or with therapeutics compared to non-hydrogel formulations (*P* = 0.0316; Fig. 4C and D). Furthermore, osteophyte size was significantly smaller in the hydrogel-containing treatment groups than the non-hydrogel groups (*P* = 0.0095; Fig. 4C and E). Collectively, intra-articular injection of PEG-4MAL hydrogel with or without DEX-loaded nanoparticles attenuated load-induced cartilage damage and osteophyte formation in the PTOA mouse model.

Our findings suggest that completely synthetic PEG-4MAL hydrogels function as a mechanical pillow that can withstand daily mechanical loads and attenuate progression of PTOA. We anticipate that such synthetic formulations could be a potential substitute for natural hydrogels, such as hyaluronic acid [52]. The mechanism of action still needs to be examined. The hydrogel may help to distribute stresses from the point of joint contact across a wider surface area in the joint. Alternately, the hydrogel may act to effectively ‘thicken’ the articular cartilage, which would reduce stresses within the tissue. Finally, the hydrogel may provide lubrication at the joint surface. In future studies, direct comparison to hyaluronic acid-based lubricants should be examined.

In addition to the hydrogel’s protective pillow-like effects in the joint space, PEG-4MAL hydrogels are inert and biocompatible. PEG-4MAL hydrogels have been shown to be cytocompatible across a large number of cell types *in vitro*, including stem cells [53], and those relevant to the musculoskeletal system [34]. PEG-4MAL is associated with safe *in vivo* use because of minimal cytotoxicity and inflammatory profiles [33, 54]. Therefore, cytotoxicity and non-biocompatibility are not anticipated issues with PEG-4MAL hydrogels in the knee joint. In addition, the location of biodegraded excipients of the hydrogel in the knee joint is unknown. However, we suspect that the degraded products will be eliminated through the lymphatic drainage from the synovium in the joint [55], the mechanism used to clear macromolecules from the synovial cavity [56]. Future studies will determine the drainage and localization of degradation products in PTOA mouse models. In summary, the PEG-4MAL hydrogel provided a safe, facile approach for an intra-articular therapy of PTOA.

In our studies, regardless of formulation, DEX did not attenuate PTOA. A limitation of the study was unequal drug dose across the formulations, i.e. hydrogel, NPs or bolus. The DEX dose in the bolus injection was higher than the dose in the nanoparticles and hydrogel treatments. We hypothesized that a high dose of bolus drug would be required to maintain the therapeutic concentration over prolonged time, whereas a controlled release formulation, even with a lower dose of drug, would release slowly and maintain the drug concentration for a longer period of time. Even with the bolus injection of DEX at a slightly higher than the clinical concentration, no attenuation of load-induced OA was observed [43]. Therefore, future studies need to further investigate this hypothesis by comparing higher doses of DEX in nanoparticles compared to bolus drug at the clinical dose. Previous approaches using intra-articular injections of corticosteroids with or without hydrogels attenuated surgically induced OA [42]. The lack of response to DEX may be attributed to the severity of load-induced OA and the mechanism of corticosteroids. A single bout of cyclic tibial compression at 9.0N-induced cartilage erosion extending to the tidemark after 2 weeks [18]. This cartilage erosion represents more advanced or severe stages of OA and occurs more rapidly than surgical models [46]. Although corticosteroids are an OARSI-recommended treatment for knee OA [57], corticosteroids are most effective in treating milder, but not severe, forms of OA [58]. Alternatively, the immune response in this non-invasive load-induced model of OA may differ from other forms of OA and affect the therapeutic release. The primary biochemical action of corticosteroids is the local suppression of inflammation, which needs to be explored further.

## Conclusion and future directions

In summary, we developed an on-demand hydrogel-based ‘mechanical pillow’ that maintained its mechanical properties in response to high levels of cyclic compression. The hydrogel system attenuated load-induced OA but dexamethasone did not. The efficacy with other therapeutics to inhibit OA progression needs to be examined. For load-induced OA the PEG-4MAL hydrogel system may be more effective in combination with other therapeutics compared to DEX. In the future, other targeted anti-inflammatory inhibitors should be examined for efficacy and synergy with the mechanical pillows. Our hydrogels maintained mechanical properties after cyclic compression, with minimal particles release up to strains that occur during normal daily activity. Further work is necessary to determine whether the hydrogel needs to withstand forces induced by tibiofemoral contact. In addition, the duration of our *in vivo* studies was 2 weeks, based on the stability of the hydrogels over 16 days for the swelling studies. The ultimate goal is to provide long-term stability in humans for 3–5 months, which is the repeat dosing time for current corticosteroid injections [59]. Future work will focus on extending the *in vivo* stability of the hydrogel system and long-term retention of therapeutics. Collectively, we demonstrated the development and characterization of an injectable, synthetic PEG-4MAL hydrogel-based mechanical pillow that can co-deliver therapeutic formulations of interest and attenuate OA-like symptoms in PTOA.
